# Whole-Genome Sequencing of the Entomopathogenic Fungus *Fusarium solani* KMZW-1 and Its Efficacy Against *Bactrocera dorsalis*

**DOI:** 10.3390/cimb46100688

**Published:** 2024-10-17

**Authors:** Junfu Yu, Mehboob Hussain, Mingqi Wu, Chunlan Shi, Shini Li, Yuanxia Ji, Sikandar Hussain, Deqiang Qin, Chun Xiao, Guoxing Wu

**Affiliations:** 1 State Key Laboratory for Conservation and Utilization of Bio-Resources in Yunnan, Yunnan Agricultural University, Kunming 650201, China; junfu_yu@163.com (J.Y.); mehboobhussain413@gmail.com (M.H.); shiclan22@163.com (C.S.); lishini06@163.com (S.L.); peakqin@163.com (D.Q.); 2 Graduate Department, Yunnan Agricultural University, Kunming 650201, China; 2008051@ynau.edu.cn; 3 Department of Agro-Environmental Science, Obihiro University of Agriculture & Veterinary Medicine, Obihiro 080-8555, Hokkaido, Japan; sikandarhussain514@gmail.com

**Keywords:** *Fusarium solani* KMZW-1, whole-genome sequencing, entomopathogenic fungus, *Bactrocera dorsalis*

## Abstract

*Fusarium solani* KMZW-1 is recognized for its potential as a biocontrol agent against agricultural and forestry pests, particularly due to its compatibility with integrated pest management (IPM) strategies. This study aimed to investigate the complete genome of *F. solani* KMZW-1 and assess its pathogenicity against *Bactrocera dorsalis*. Whole-genome sequencing revealed a genome size of 47,239,278 bp, comprising 27 contigs, with a GC content of 51.16% and fungus identified as *F. solani* KMZW-1. The genome completeness was assessed as 97.93% using BUSCO analysis, the DFVF sequence identifier was *Fusarium* 0G092560.1, and AntiSMASH analysis identified 35 gene clusters associated with secondary metabolite biosynthesis, providing insights into the genetic basis of its pathogenic mechanisms and biocontrol potential. Comparative genomic analysis found 269 unique genes for *F. solani* KMZW-1, and collinearity analysis exhibited a high degree of synteny with *Fusarium solani-melongenae*. The pathogenicity of *F. solani* KMZW-1 was assessed using concentrations ranging from 1 × 10^4^ to 1 × 10^11^ conidia/mL. Higher concentrations (1 × 10^10^ to 1 × 10^11^ conidia/mL) resulted in significantly increased cumulative mortality rates of *B. dorsalis* adults compared to the control group. Notably, the pathogenicity was higher in male adults than in females. Probit analysis yielded LC_50_ (50% lethal concentration) values of 5.662 for female and 4.486 for male *B. dorsalis* adults. In summary, *F. solani*, KMZW-1 exhibits strong insecticidal activity against *B. dorsalis* and shows potential as a biocontrol agent with IPM strategies. These findings provide robust genomic evidence supporting the use of *F. solani* KMZW-1 in managing against *B. dorsalis* populations.

## 1. Introduction

*Fusarium solani* KMZW-1 is a well-recognized entomopathogenic fungus belonging to the family Tuberculariaceae, order Hypocreales. Recently, biological control has garnered increased attention for managing agricultural insect pests due to its environmental friendliness. Biocontrol agents, such as *F. solani* KMZW-1, have become a focal point for experts seeking sustainable pest management solutions. *Bactrocera dorsalis* (Diptera, Tephritidae), commonly known as the oriental fruit fly, is a significantly destructive and persistent pest affecting over 250 host plants, as documented [1]. Effective control of *B. dorsalis* is crucial for minimizing crop damage and financial losses.

Previous researches have shown the insecticidal potential of biocontrol agents. For example *Metarhizium anisopliae* (Hypocreales, Clavicipitaceae) and *Beauveria bassiana* (Hypocreales, Cordycipitaceae) have demonstrated promising results in controlling *Bactrocera cucurbitae* (Diptera: Tephritidae). Hamzah et al. (2021) reported mortality rates of 73.43 and 59.72% for contact and oral application respectively. They further reported the increase in pathogenicity of both fungus over increased concentration and time interval [1]. Similarly, Li et al. (2024) screened six strains of *B. bassiana* and found a mortality rate of 90.67% for adults of oriental fruit fly with the most active strain B4 [2]. These studies provide a theoretical basis for exploring new biocontrol agents against *B. dorsalis,* highlighting the need for further research into the pathogenicity of *F. solani* KMZW-1.

In recent years, advancements in biotechnology have provided new insights into the success of biological control. Genomic sequencing and comparative genomics analyses have become focal points in studying the biocontrol potential of entomopathogenic fungi, offering crucial insights about their biological characteristics and potential applications [3]. For instance, genetic data-based fungal co-expression network analysis of the entomopathogenic fungus *B. bassiana* has revealed the infection-associated modules and host–pathogen interaction related to its biocontrol potential against insect pests [4]. Similarly, *B. bassiana* exhibits an endo-β-1,3-glucanase (BbEng1) that helps in pathogenicity by accelerating fungal mycosis through decreasing glucan PAMPs in the cell wall of the host insect [5]. These findings offer a deep understanding of the genetic characteristics, pathogenic mechanisms, and biocontrol potential of the entomopathogenic fungus. 

Whole-genome sequencing has emerged as a powerful tool for understanding the genetic basis of biological traits and behaviors in various organisms. The complete genome analysis of *L. attenuatum* Strain Lec8 revealed a genome size of 32.38 Mb, containing 9531 genes, including those encoding proteins related to biosynthetic pathways, which suggested strong entomopathogenic potential [6]. Similarly, the genome sequencing of *F. solani-melongenae* (CRI 24-3) revealed a 49.6 Mb chromosome-level draft genome containing 15,374 putative coding genes, Contigs N50, 4,496,268 (bp), and GC content was 50.7%. It also showed relatively high numbers of virulence factors, such as carbohydrate-active enzymes (CAZymes), pathogen–host interaction (PHI) proteins, and terpene synthases (TSs) [7]. These findings provide significant insights on the molecular basis of the fungus, though further research is needed for exploring its pathogenic potential. Whole-genome sequencing may explore the genetic basis of *F. solani* KMZW-1 as a biocontrol agent. 

Fruit flies pose a major threat to a wide range of fruits and vegetables globally from tropical to subtropical environments due to their polyphagous nature [8,9]. *B. dorsalis*, in particular, is a significant threat to horticultural crops in China and neighboring regions. Biological control of many insect pests, including fruit flies, frequently employs entomopathogenic microorganisms [10,11,12,13]. This study aims to explore the entomopathogenic potential of *F. solani* KMZW-1 against *B. dorsalis* by utilizing complete genome sequencing and comparative genomics. Additionally, we compared its pathogenicity potential against different sexes of *B. dorsalis*. 

## 2. Materials and Methods

### 2.1. Fusarium solani KMZW-1 Culture and Identification

Adult *Boettcherisca peregrina* flies infected with a wild strain of fungus were collected from a pig farm at Yunnan Agricultural University, Animal Science Institute, Kunming City. Newly emerged 4-day-old *B. peregrina* flies, disease-free and of uniform size, were individually placed in small rearing cages and provided with ample water and white sugar. They were maintained under controlled conditions of 25 ± 0.5 °C and a (12L:12D) light cycle. The wild strain of fungus was isolated from naturally infected *B. peregrina* flies and cultured on Potato Dextrose Agar (PDA). The third strain was subsequently reintroduced to the *B. dorsalis* flies. The strain was propagated and expanded on PDA medium at 26 °C and a pH of 6.0 following Usman et al. (2021) with slight modifications [14]. 

The DNA of the fungal strain was extracted using the Ark DZ306-02 Fungal DNA Mini Kit (Tsingke Biotechnology Co., Ltd., Beijing, China). PCR amplification was conducted utilizing the universal primers ITS1 (5′-TCCGTAGGTGAACCTGCGG-3′) and ITS4 (5′-TCCTCCGCTTATTGATATGC-3′), which generated approximately 700 bp. The amplified products were subsequently analyzed by agarose gel electrophoresis to assess band size accuracy, band singularity, and the presence of any smearing. Upon confirming the expected band size and quality of the PCR products, the target bands were excised and purified. The purified DNA was then subjected to Sanger sequencing. Sequencing results were assembled using ContigExpress software (Version 9.1), trimming off the inaccurate ends. The assembled sequences were subjected to batch BLASTn [15] (Basic Local Alignment Search Tool, version 2.13, available at https://blast.ncbi.nlm.nih.gov/Blast.cgi, URL (accessed on 30 August 2024) to align against the nucleotide database. This BLASTn search, conducted against the latest version of the nucleotide (nt) database, facilitated the retrieval of accession numbers and the species identification and annotation of homologous sequences. 

### 2.2. Insect Collection and Rearing

The population of *B. dorsalis* was collected from fallen fruits in a mango orchard located in the suburb of Kunming (latitude 23°36′ N, longitude 101°58′ E, altitude 460.97 m). Infested fruits were placed on sandy soil until hatching and pupation. After pupation, the pupae were collected and moved indoors. Upon adult emergence, the flies were provided with a diet consisting of 10% yeast extract, 5% peptone, 30% white sugar, 15% agar, and plain water. For egg collection, mating females were allowed to oviposit in plastic bottles lined within stint fruit juices coating the inner walls. The eggs were then transferred using 100-mesh nylon netting to fresh keeping boxes containing larval feed composed of 10% yeast extract, 5% peptone, 30% white sugar, 10% agar, and 40% wheat bran. Larvae were allowed to mature and pupate in the soil. The emerged adults from the third generations were maintained in rearing cages (30 cm × 30 cm × 30 cm); indoor environmental conditions were controlled at a temperature of 25 ± 1 °C, relative humidity of 60 ± 10%, and photoperiod of 14L:10D. 

### 2.3. Pathogenicity Test of Fusarium solani KMZW-1 Against Bactrocera dorsalis

#### 2.3.1. Conidial Suspension Preparation

The fungal strain *F. solani* KMZW-1, stored at −80°, was used for fresh culture. A 100 μL aliquot of the fungal suspension was evenly spread onto the PDA solid medium containing kasugamycin using a disposable spreader. The plates were then incubated at a constant temperature of 26 °C in an incubator for subsequent use. Initially, a sterile 0.05% (*v*/*v*) Tween-80 solution was prepared by mixing 200 μL of Tween-80 solution and 400 mL of deionized water; then it was sterilized by autoclaving at 115 °C for 30 min. The conidial suspension derived from shaking flask cultures was filtered through a four-layer sterile gauze to eliminate the mycelial fragments. The resultant filtrate was then distributed into 50 mL centrifuge tubes and centrifuged at 4 °C at 12,000× *g* rpm for 10 min. The supernatant was discarded, and the conidial pellet re-suspended in an adequate volume of the aforementioned 0.05% (*v*/*v*) Tween-80 solution. To ensure homogeneity, the suspension was mixed vigorously in a vortex mixer for 3 min. Concentration determination of the conidia was performed by using a hemocytometer featuring a 25 × 16 grid pattern. Each sample underwent triplicate counts to enhance the precision. The targeted concentrations were 1 × 10^4^, 1 × 10^5^, 1 × 10^6^, 1 × 10^7^, 1 × 10^8^, 1 × 10^9^, 1 × 10^10^, and 1 × 10^11^ conidia per milliliter. The calculation for the conidial suspension was performed according to the following equation:

Conidial suspension = (total count from five squares) × (dilution factor) × 5 × 10^4^ (conidia/mL).

#### 2.3.2. Bioassays

The experiment utilized the hit-and-trial method. A small manual sprayer was employed to uniformly spray the conidial suspensions, adjusted to the distinct concentrations, onto the flies until their cuticles appeared to be visibly moistened. Mortality was monitored every 24 h for 12 consecutive days. All were treated according to Zhang et al. (2021) with slight modifications [16]. To ensure equal conidial doses were applied topically, we continuously shook the sprayer and 5 mL of the conidial suspension per replicate used for each concentration. For the control treatment, sterile 0.05% (*v*/*v*) Tween-80 solution was sprayed. Insects were placed in rearing chambers at a temperature of 26 °C, relative humidity of 85%, and a 12:12 h photoperiod. Treated insects were given adult feed (liquid cultured medium) and water throughout the experiment. Each treatment group consisted of 30 insects, and all experiments were performed in triplicate. Male and female adults were separated for the experimentation, with no age specificity. The dead cadavers of flies were observed for fungal outgrowth (infection) under the light microscope (BA310-T, Motic®, Mack Audi industrial group Co., Ltd., Beijing, China).

#### 2.3.3. Data Analysis

The cumulative mortality rate of *B. dorsalis* was statistically analyzed using Microsoft Office Excel 2021. The LC50 values were calculated using Probit analysis, and the results were plotted on a logarithmic scale (base 10). Graphs were generated using GraphPad Prism (USA, 8.3.0538).

### 2.4. Complete Genome Sequencing, Assembly, and Annotation

#### 2.4.1. Genome Sequencing

The genome sequencing process followed the standard protocol provided by Oxford Nanopore Technologies (ONT, Oxford, UK), encompassing sample quality assessment, library construction, library quality detection, and library sequencing conducted by the Biomarker Technologies Co., Ltd. (Beijing, China). The library construction process included the following steps as genomic DNA extraction and quality control, fragment recovery, and library construction using an SQK-LSK109 sequencing kit. SpotON flow cell R9.4.1 FLO-MIN106 was used to load the library and to sequence it on MinION Mk1C for 72 h. High-quality genomic DNA was extracted, and its purity, concentration, and integrity were checked using Nanodrop spectrophotometry, Qubit fluorometry, and 0.35% agarose gel electrophoresis. The BluePippin automatic nucleic acid recovery system was used to recover the large DNA fragments.

(a) DNA damage repair and end repair were performed, followed by magnetic bead purification;
(b) DNA fragments were ligated with adapters, followed by another round of magnetic bead purification;
(c) Qubit fluorometry was used for library quantification;
(d) Sequencing was performed using PromethION48 sequencer (ONT, Oxford, UK).

#### 2.4.2. Genome Assembly

The filtered subreads were assembled using the NECAT tool [17], and the initial assembly results were corrected using Racon software v1.4.3 [18] with the filtered subreads. 

After completing the assembly of the *F. solani* KMZW-1 genome, its completeness was assessed using BUSCO (Benchmarking Universal Single-Copy Orthologs, v3.0.2). The analysis employed the fungi_odb9 database [19], which includes 29,000 single-copy orthologous genes from 185 fungal species. The completeness of the genome assembly was determined by calculating the percentage of complete single-copy genes identified in the *F. solani* KMZW-1 genome sequence compared to the total number of single-copy genes in the database.

#### 2.4.3. Genome Predictions 

The software LTR_FINDER v1.05 [20], MITE-Hunter (UNIX program pipeline) [21], RepeatScout software v1.0.5 [22], and PILER-DF software v2.4 [23] were used to construct the repeat sequence database. The database was classified using the PASTE Classifier database [24] and merged with the Repbase tool [25] to form the final repeat database. Repeat Masker software v4.0.6 [26] was then used to predict the repeat sequence. 

Gene-structure prediction primarily involves ab initio prediction, homology-based protein prediction, and transcriptome data prediction; the ab initio prediction tools used were Genscan [27], Augustus v2.4 [28], Glimmer HMM v3.0.4 [29], Gene ID v1.4 [30], and SNAP (version 2006-07-28) [31]. Homology-based protein prediction was performed using GeMoMa software v1.3.1 [32]. Predicted results were integrated using EVM software v1.1.1 [33] and refined using PASA v2.0.2. tRNA genes were predicted using tRNAscan-SE v1.3.1 [34], and drRNA genes were predicted using Infernal software v1.1.1 [35]. 

Genomic scanning was performed using software GenBlastA v1.0.1 [36] to filter the predicted functional genes. We used the predicted protein sequences and the protein sequences collected in the Swiss-Prot database; through software GenBlastA comparison, we searched for homologous gene sequences (likely genes) on the genome. Subsequently, GeneWise v2.2.0 [37] was utilized to identify immature and frame-shift mutations, followed by analysis of presumed candidates. AntiSMASH v6.0.0 [38] was employed to predict the secondary metabolite gene clusters.

#### 2.4.4. Genome Protein Annotations

The predicted proteins were subjected to a BLAST online web search (https://blast.ncbi.nlm.nih.gov/Blast.cgi, URL (accessed on 14 October 2024) [39] against various databases: Nr [40], Swiss-Prot [41], TrEMBL [42], KEGG [42], and KOG [43]. GO annotations were performed using annotations using Blast2GO software v6.0 [44] based on the result of BLAST searches. Pfam domains were identified using HMMER v3.3.2 [45]. In addition, pathogenicity-related proteins were determined using the CAZy [46], TCDB [47], PHI [48,49], CYPED, and DFVF databases [50], and others. Secreted proteins were detected using SignalP v4.0 [51], and transmembrane proteins were filtered by TMHMM software v2.0 [52] to isolate candidate secreted proteins. Effector proteins among the P secreted proteins were predicted utilizing EffectorP [53].

#### 2.4.5. Comparative Genome Analysis 

Comparative genome analysis was conducted to comprehensively understand the distinctions between *F. solani* KMZW-1 and other genomes (GenBank accession number: *Fusarium solani-melongenae*: GCA_023101225.1, *Fusarium solani*: GCA_023522795.1, *Fusarium oxysporum*: GCA_013085055.1, *Fusarium* sp. Ph1: GCA_025433565.1, *Fusarium* sp. LHS14.1: GCA_025433615.1). Phylogenetic and molecular evolutionary analyses were performed using MEGA software version 11 [54]. OrthoFinder software (version 2.5.5) was employed to identify single-copy orthologous genes and classify gene families. The analysis was performed using “-M msa” for the multiple sequence alignment method and “-S Diamond” for the sequence similarity search tool [55]. Genomic collinearity analysis was performed using TBtools v2.025 [56], with visualization of the results conducted through NGenomeSyn [57]. 

## 3. Results

### 3.1. Pathogenicity Test of Fusarium solani KMZW-1 at Different Concentrations

**Figure 1:**
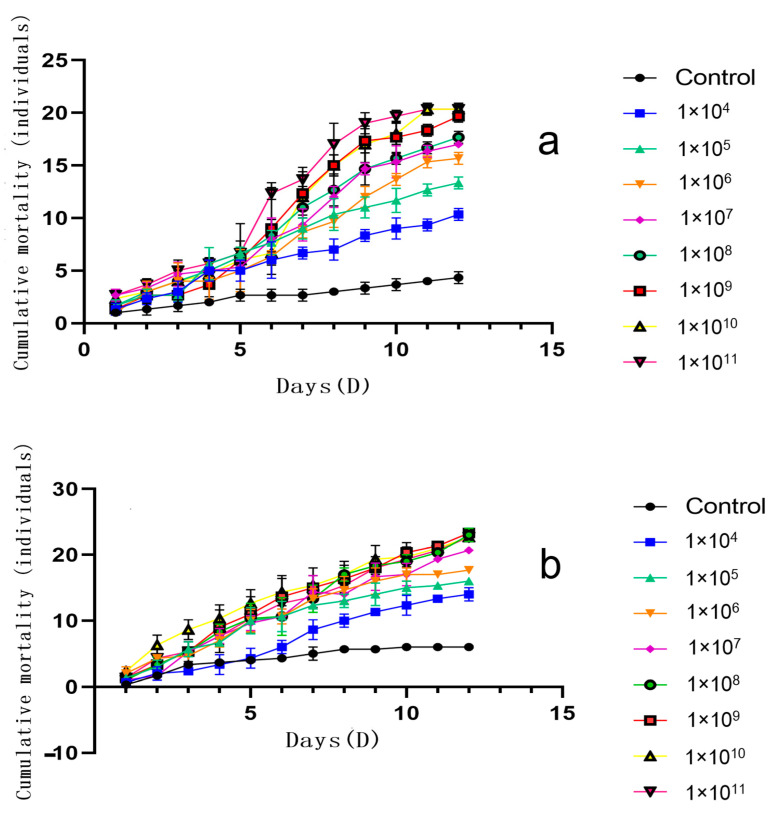
The variation in cumulative mortality count under different treatments for *Bactrocera dorsalis* adults over 12 days. Note: Figure (**a**) shows the cumulative mortality of female *Bactrocera dorsalis* adults; Figure (**b**) shows the cumulative mortality of male *Bactrocera dorsalis* adults. Error bars = SD, data analyzed by non-linear regression.

The cumulative mortality of *B. dorsalis* adults infected with varying concentrations of *F. solani* KMZW-1 conidial suspensions (female adults in Figure 1a, male adults in Figure 1b) over time is given in Figure 1. The lines represent different concentrations of *F. solani* KMZW-1 conidial suspensions ranging from 1 × 10^4^ conidia/mL to 1 × 10^11^ conidia/mL, while the control group represents the population not infected with the fungus. The cumulative mortality increases over time in all treatment groups, indicating that as time progresses, more *B. dorsalis* adults were infected. The difference between the control group and the treatment groups indicates that fungal infection was the main cause of increased mortality of *B. dorsalis*. Regarding the time effect, in the early stages (e.g., first 5 days), the differences in mortality rates among different concentration treatment groups were not significant. However, as the experiment progressed, the mortality rate in the high concentration treatment groups becomes significantly higher than that in the low concentration treatment groups. This indicates that *F. solani* KMZW-1 requires some time to proliferate within the host and cause death. By comparing the insecticidal rates of different high concentrations against female and male adults of *B. dorsalis*, it is evident that *F. solani* KMZW-1 has a significantly higher insecticidal effect against male adults. At each concentration gradient, the insecticidal rate for male adults is approximately 10% higher than that for female adults.

**Figure 2:**
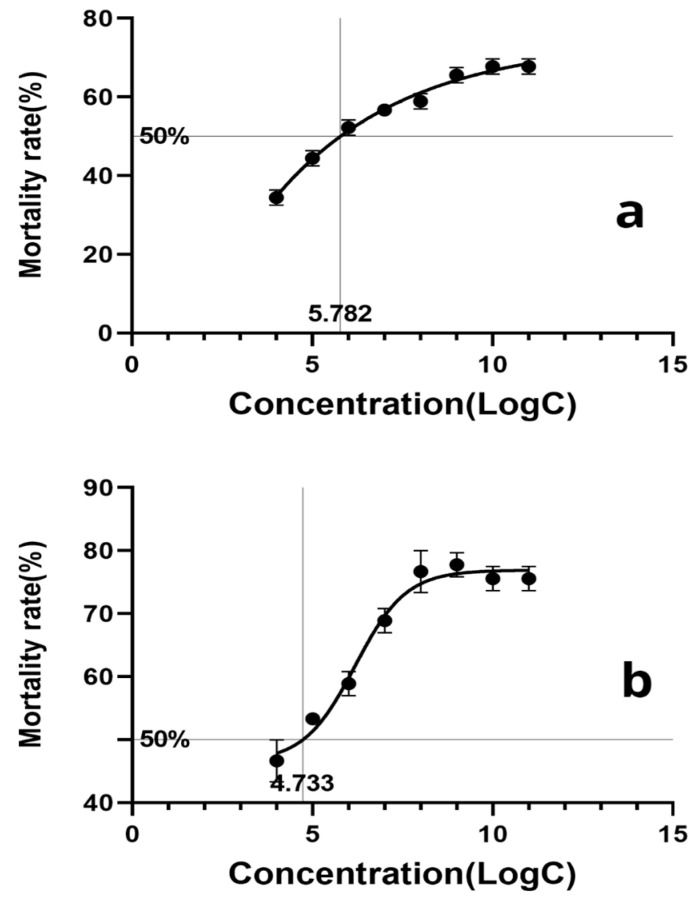
The calculation of lethal concentration for *Bactrocera dorsalis* adults over 12 days. Note: (**a**) The calculation of lethal concentration for female *Bactrocera dorsalis* adults; (**b**) the calculation of lethal concentration for male *Bactrocera dorsalis* adults. Error bars indicate significant differences among different replications of each treatment.

**Figure 3:**
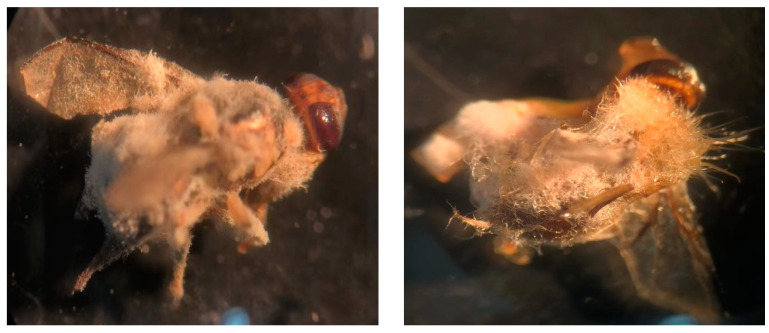
Dead cadavers of *Bactrocera dorsalis* adults with fungal outgrowth.

Based on the calculation, the LogLC50 of *F. solani* KMZW-1 against adult *B. dorsalis* females was determined as 5.662 and against male adults as 4.486 (Figure 2). A significant increase in the mortality was observed as the conidial concentration increased from LogC4 to LogC9. Particularly at LogC6 and higher concentrations, the mortality rate rapidly reaches high levels, suggesting a strong lethal effect of *F. solani* KMZW-1 against *B. dorsalis*. The dead cadavers of *B. dorsalis* observed for fungal outgrowth under the light microscope to confirm the fungal infection (Figure 3). 

### 3.2. ITS-Based Identification of Fusarium solani KMZW-1

Through strain identification, the KMZW-1 strain is identified as *F. solani* with an identification value of 569/572 (99.48%), and the blast alignment score is 1038. The annotation of the alignment result is *F. solani* genes for 18S rRNA, ITS1, 5.8S rRNA, ITS2, 28S rRNA, partial and complete sequence, isolate: FKI-6853. Based on these results, the strain is identified as *F. solani* KMZW-1, commonly known as *Fusarium*.

### 3.3. Genome Assembly Results

A total of 6,424,650,682 bp of raw data was obtained using Nanopore third-generation sequencing (NCBI Accession number PRJNA1171171). After filtering for adapters, short fragments, and low-quality data, a total of 6,424,650,682 bp of clean reads were obtained, with a sequencing depth of 136X. The genome assembly of *F. solani* KMZW-1 resulted in 27 contigs with a total length of 47,239,278 base pairs (bp). Key metrics include an N50 of 2,751,789 bp and an N90 value of 1,018,923 bp, indicating a higher proportion of larger contigs in the assembly and overall good contiguity. The GC content of the genome was 51.16%, providing insights into its genetic composition (Table 1). Importantly, the assembly process gained closure without any gaps showing in the genome assembly. This achievement is crucial for subsequent analyses, such as gene structure analysis, functional annotation, and evolutionary analysis. The statistical results of the gene assembly were as follows in Table 1.

### 3.4. BUSCO Evaluation

We utilized the fungi_odb9 database in BUSCO [19], which contains 290 conserved core genes for fungi to evaluate the completeness of our fungal genome assembly using BUSCO (v2.0). A total of 284 complete BUSCO genes were identified in our assembly, resulting in a genome completeness evaluation of 97.93%. Among these, 281 genes were identified as single-copy, constituting 96.90% of the total BUSCO genes assessed. The presence of these genes in a single copy showed the high accuracy and completeness of the genome assembly. Additionally, three genes were identified as multi-copy, accounting for 1.03% of total genes. These genes likely exist in multiple copies or repetitive sequences influencing the assembly process. Four genes were predicted to be incomplete, accounting for 1.38% of total genes. These incomplete genes may result from assembly gaps or missing sequences in the genome as the assembly process. Furthermore, two genes were not predicted at all, constituting 0.69% of total genes, indicating that some conserved genes were not detected during the genome assembly process (Table 2). 

### 3.5. General Database Annotations

Gene sets were annotated using different databases: GO Annotation, KEGG Annotation, KOG Annotation, Pfam Annotation, Swissprot Annotation, TrEMBL Annotation, and nr Annotation (Table 3). A total number of 13,869 genes were annotated, indicating that the majority of genes obtained at least one functional annotation, providing a solid foundation for subsequent gene function studies. For example, annotations varied across databases: GO Annotation annotated 9994 genes, KEGG Annotation annotated 3775 genes, KOG Annotation annotated 7059 genes, Pfam Annotation annotated 10,510 genes, Swissprot Annotation annotated 10,651 genes, TrEMBL Annotation annotated 13,862 genes, and nr Annotation annotated 13,867 genes. For genes with lengths in the range of 100 ≤ length < 300 bp, annotations varied from 913 (KEGG) to 3733 (All Annotated), indicating that even shorter gene fragments can obtain a certain degree of annotation. However, the genes with lengths greater than or equal to 300 bp generally received higher annotation quantities. For instance, the nr database annotated 9995 genes, indicating that longer genes are more likely to receive functional annotations.

### 3.6. GO Annotations

**Figure 4:**
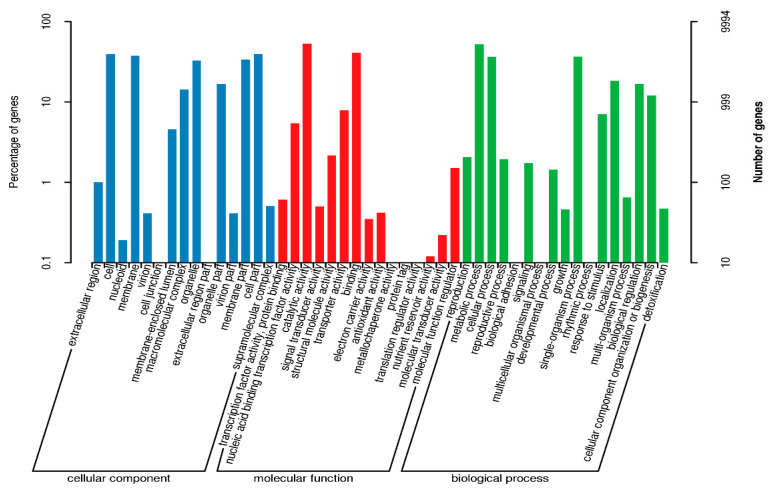
Statistics of GO annotation classification of *Fusarium solani* KMZW-1. Note: The horizontal coordinate is the content of GO classification, the left side of the vertical coordinate is the percentage of the number of genes, and the right side is the number of genes. This figure shows the gene enrichment of each secondary function of GO in the context of all genes, reflecting the status of each secondary function in this context.

The Gene Ontology (GO) annotation classification for the genes of *F. solani* KMZW-1 is given in Figure 4. Biological Process (BP) with 4444 genes, Cellular Component (CC) with 2279 genes, and Molecular Function (MF) with 7603 genes were found. Among these categories, the “metabolic process” category has the highest number of genes, with 1401 genes, followed by the “binding” category with 938 genes. Additionally, there were categories such as catalytic activity, structural molecule, cellular component, and others, with gene counts of 689, 462, 239, and 218, respectively.

### 3.7. KOG Annotations

**Figure 5:**
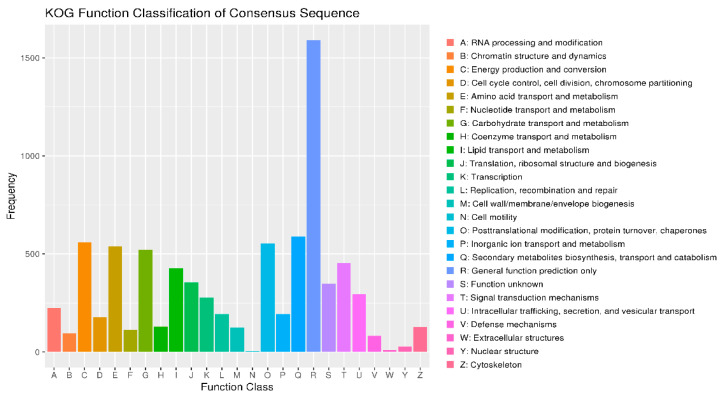
KOG functional annotation classification of *Fusarium solani* KMZW-1. Note: The horizontal coordinate is the classification content of KOG, and the vertical coordinate is the number of genes. In different functional categories, the proportion of genes reflects the metabolic or physiological bias in the corresponding period and environment, which can be scientifically explained in combination with the distribution of research objects in each functional category.

Energy production and conversion (C) contains approximately 1500 genes, the highest among all categories. This suggests that *F. solani* KMZW-1 extensive energy metabolism pathways are essential for adapting to variable environments (Figure 5). Amino acid transport and metabolism (E) included around 1000 genes, indicating specialization in amino acid metabolism crucial for protein synthesis and cell growth. Nucleotide transport and metabolism (F) has a similar number of genes as the amino acid metabolism, approximately 1000 genes, reflecting complexity in nucleotide metabolism, crucial for fundamental life activities such as DNA replication, repair, and RNA metabolism. Carbohydrate transport and metabolism (G) also comprises around 1000 genes, indicating *F. solani* KMZW-1’s ability to effectively utilize and regulate carbohydrate metabolism, crucial for energy production and carbon skeletons. Translation, ribosomal structure, and biogenesis (J) encompasses around 500 genes reflecting *F. solani* KMZW-1 activity in protein synthesis, vital for its growth and development. Replication, recombination, and repair (L) includes a moderate number of genes, indicating *F. solani* KMZW-1 mechanisms for maintaining genome stability, crucial for its long-term survival and adaptability. Secondary metabolites biosynthesis, transport, and catabolism (Q) involves relatively fewer genes but is crucial for the production of bioactive compounds in *F. solani* KMZW-1, potentially influencing pathogenic potential. Function unknown (S) encompasses genes whose functions are yet to be fully elucidated; its presence suggested unexplored functional areas in the genome of *F. solani* KMZW-1, which could include undiscovered novel functions or specific metabolic pathways.

### 3.8. KEGG Annotations

**Figure 6:**
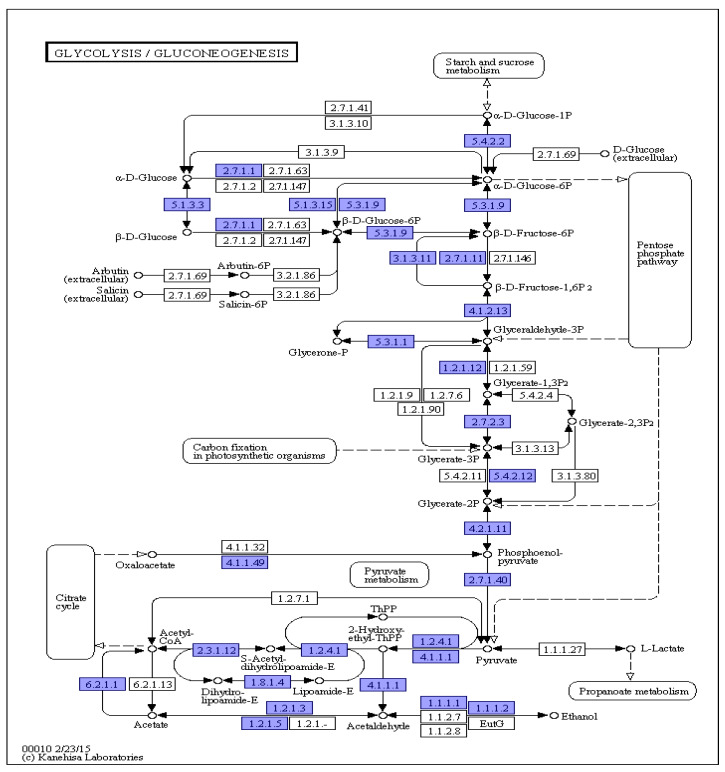
Schematic diagram of KEGG metabolic pathway results for *Fusarium solani* KMZW-1. Note: the number in the box represents the EC number of the key enzyme in the metabolic pathway, and the blue box indicates the presence of the coding gene corresponding to the enzyme in the genome.

*F. solani* KMZW-1 has the complete metabolic capability to regulate its energy production and storage (see Figure 6). Additionally, the diagram illustrates pathways related to the sugar metabolism, such as lactate fermentation and pyruvate fermentation, which may function under anaerobic conditions or specific environmental conditions.

### 3.9. Genetic Information Processing

**Figure 7:**
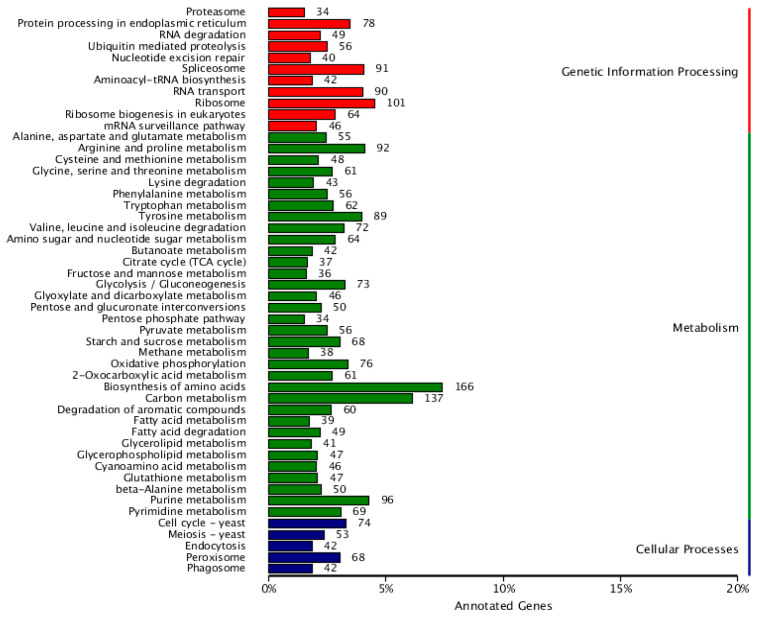
Classification statistics of KEGG annotations of *Fusarium solani* KMZW-1. Note: the vertical coordinate is KEGG secondary classification, and the horizontal coordinate is percentage.

Significant categories include “Ribosome” (101 genes, approximately 10%), “Spliceosome” (91 genes), and “mRNA surveillance pathway” (46 genes). These data indicate that *F. solani* KMZW-1 has complex protein synthesis and RNA processing mechanisms, which are crucial for its growth and adaptability (see Figure 7).

Cellular processes: Processes like, “Endocytosis” (5%) and “Phagosome” (42 genes), which involve the uptake and processing of substances within the cell, likely play a crucial role in *F. solani* KMZW-1 nutrient acquisition and defense mechanisms.

Metabolism: Categories such as “Biosynthesis of amino acids” (166 genes, approximately 16%) and “Purine metabolism” (96 genes) encompass a relatively large number of genes, indicating that *F. solani* KMZW-1 has complete pathways for amino acid synthesis, essential for cell growth, and DNA synthesis.

### 3.10. DFVF Database Annotation

**Figure 8:**
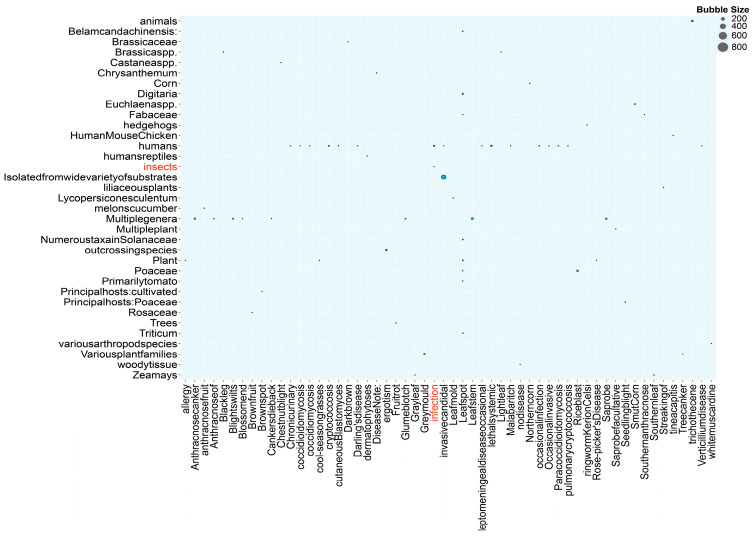
DFVF annotation results of *Fusarium solani* KMZW-1.

The Database of Known Fungal Virulence Factors (DFVF), available online at http://sysbio.unl.edu/DFVF, URL (accessed on 24 June 2024), represents a comprehensive resource that aggregates data on the 2058 genes associated with pathogenicity, derived from a diverse collection of 228 fungal strains encompassing 85 genera. In the present annotation endeavor, a total of 3054 genes encoding factors pertinent to fungal virulence have been meticulously annotated. Referencing Figure 8, the DFVF annotation bubble plot highlights that the gene designated by the sequence identifier *Fusarium* 0G092560.1 exhibited strong virulence activity specifically relevant to the infection of insect hosts, which suggests its insecticidal potential against insect hosts.

The prediction of gene clusters indicates a total of 35 gene clusters distributed across 15 different genomic contigs using antiSMASH analysis, with a length range from 40,236 to 90,146 bp and an average length of approximately 46,000 bp. The predicted pseudogene results in the studied genome reveal the presence of seven pseudogenes, with a total length of 1463 bp (see Supplementary Materials). 

### 3.11. Comparative Genomic Analysis

**Figure 9:**
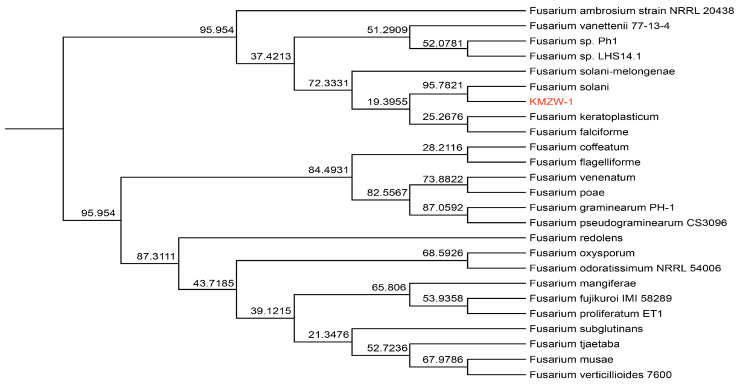
Phylogenetic tree of *Fusarium solani* KMZW-1 with different strains.

KMZW-1 has the closest phylogenetic relationship with *F. solani* in the phylogenetic tree, with a bootstrap value of 95.7824, indicating a high level of genetic similarity and the possibility that they share many evolutionary traits and ecological functions (see Figure 9). It is also relatively close to the strain *F. solani-melongenae*, with a bootstrap value of 72.3331. The bootstrap value between *F. solani* KMZW-1 and *F. keratoplasticum* is 25.2676, suggesting that although they belong to the same *Fusarium* genus, they have significant genetic differences and evolutionary divergence. Furthermore, the bootstrap values between *F. solani* KMZW-1 and *F. falciforme* and *F. coffeatum* are 28.2116 and 19.3965, respectively, further indicating that these species have progressively diverged in the evolutionary process, and their phylogenetic relationship is relatively distant. In comparison, *F. solani* KMZW-1 is more distantly related to *F. graminearum* (bootstrap value 87.0592) and *F. poae* (bootstrap value 73.8822), showing greater genetic differences. The bootstrap value of 68.5926 with *F. oxysporum* indicates significant evolutionary divergence between *F. solani* KMZW-1 and this group of species. These data suggest that while KMZW-1 has a close evolutionary relationship with *F. solani*, it has shown different divergence pathways with other species during the evolutionary process, possibly reflecting its adaptive evolution in different ecological environments. 

**Figure 10:**
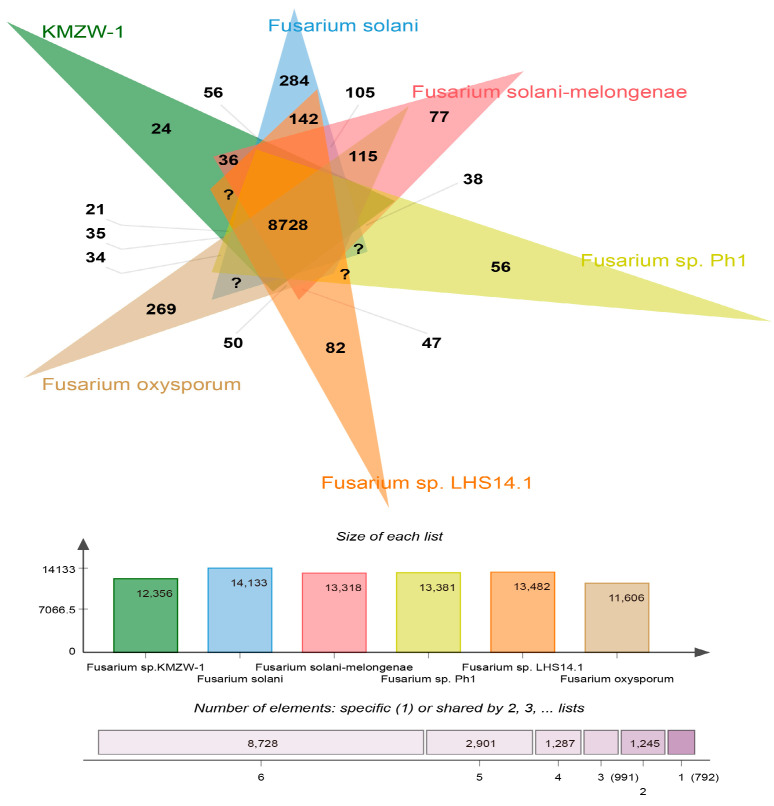
Comparative analysis of homologous genomes.

Genomic comparison using OrthoFinder reveals distinct and shared genetic characteristics of *F. solani* KMZW-1 in relation to other *Fusarium* strains (Figure 10). Notably, *F. solani* KMZW-1 contains 269 unique genes, underscoring its genetic distinctiveness and potential for unique ecological adaptations. The strain shares 142 genes with *F. solani*, indicating a closer evolutionary relationship with this species. In contrast, it shares fewer genes with *F. solani-melongenae* (77 genes), *Fusarium* sp. Ph1 (115 genes), *Fusarium* sp. LHS14.1 (82 genes), and the least with *F. oxysporum* (56 genes), suggesting varying degrees of genetic divergence among these species. The core genome shared across all analyzed strains consists of 8728 genes, likely representing essential functions conserved within the *Fusarium* species. These findings highlight the unique genetic profile of *F. solani* KMZW-1 and provide insights into its evolutionary relationships with other *Fusarium* strains.

**Figure 11:**
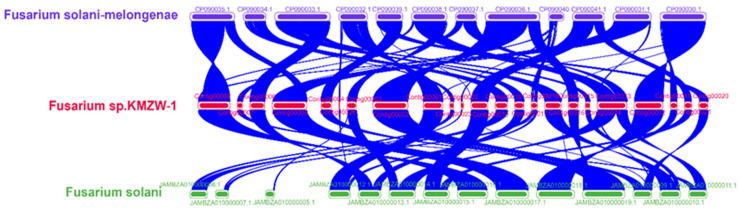
Collinearity analysis between the genomes of *Fusarium solani* KMZW-1, *Fusarium solani-melongenae*, and *Fusarium solani*.

Figure 11 presents a synteny analysis of the genomes of *F. solani* KMZW-1, *F. solani-melongenae*, and *F. solani*. The blue lines depict shared syntenic regions among the three genomes, illustrating a high degree of genomic similarity and conservation throughout their evolutionary histories. This conservation likely reflects shared biological functions or ecological adaptations among these species. Notably, the synteny between *F. solani* KMZW-1 and *F. solani-melongenae* is more extensive, indicating a higher degree of similarity across multiple genomic regions. This suggests that these strains may have been subjected to similar selective pressures or ecological adaptations. In contrast, the synteny with *F. solani* is relatively sparse, pointing to differences in their evolutionary trajectories. These differences may arise from divergent selective pressures, genome rearrangements, or specific functional evolutionary adaptations, further underscoring the genomic uniqueness of *F. solani* KMZW-1. These findings provide important genomic evidence for understanding the evolutionary relationships, as well as the ecological and functional characteristics, of these *Fusarium* strains.

## 4. Discussion

In recent years, whole-genome sequencing has emerged as an important tool for elucidating the genetic characteristics and biological mechanisms of biocontrol fungal strains [58]. Comparative analyses have revealed distinct genomic features among these strains, including variations in GC content, numbers of coding genes, and specific gene clusters. In our study, whole-genome sequencing revealed a genome size of 47,239,278 bp, comprising 27 contigs, with a GC content of 51.16%. The genome completeness was assessed as 97.93% using BUSCO analysis, the DFVF sequence identifier was *Fusarium* 0G092560.1, and antiSMASH analysis identified 35 gene clusters associated with secondary metabolite biosynthesis, providing insights into the genetic basis of its pathogenic mechanisms and biocontrol potential. Our findings align with previous whole-genome sequencing studies on different organisms. For example, the whole-genome sequence of entomopathogenic fungus *B. bassiana* JEF-350 revealed a total of 34,655,292 Illumina reads (5,232,949,092 bases), 49.61% G1C content, eight assembled contigs, and 22,202,500 filtered Illumina reads. Annotation of protein-coding genes and functional annotation showed that there were 28,999 and 17,771 exons and introns, respectively, while a total of 11,225 protein-coding genes were predicted. Total genes annotated for transport and metabolism related to amino acids, nucleotides, coenzymes, carbohydrates, lipids, and inorganic ions in the eggNOG classification were 1593 [59]. A 49.6 mb chromosome-level draft genome containing 15,374 putatively coding genes was obtained with a GC content of 50.7% and 12 scaffolds by sequencing the pathogenic fungus *F. solani-melongenae* (CRI 24-3) using third-generation and next-generation sequencing techniques [6]. In another study by Lee et al. (2024), a hybrid assembly approach was used to study the whole-genome sequencing of *B. bassiana* strain KNU-101. The genome analysis revealed comprehensive insights into its genetic makeup. The genome sequencing of strain KNU-101 showed a maximum scaffold length of 10,066,884 bp with a GC content of 49.49%, 26 contigs and 1,822,896 reads (9,827,426,196 bp), with an N50 read length of 13,949 bp through nanopore sequencing [60]. Similarly, the genome analysis of *B. pseudobassiana* strain RGM 2184 resulted in 114 genes encoding for extracellular enzymes and four biosynthetic gene clusters reported as producers of insecticidal and bactericidal factors, such as oosporein, beauvericin, desmethyl-bassianin, and beauveriolide. Comparative genomic analysis revealed that 65–95% of these genes are *Beauveria* genus-specific. Metabolic profiling of supernatant extracts from RGM 2184 cultures exhibited secondary metabolites, such as beauveriolide, oosporein, inflatin C, and bassiatin, which were the main factors involved in its insecticidal activity [61]. 

This study performed comprehensive functional annotation of the genome of *F. solani* KMZW-1, including annotations from GO, KEGG, KOG, and DVDF databases. The results showed 9994 GO annotations, 3775 KEGG annotations, 7059 KOG annotations, and 3054 DVDF annotations. In a similar study, Iwanicki et al., (2022) conducted genome sequencing of *Metarhizium humberi* (Hypocreales, Clavicipitaceae) in order to obtain its genomic signatures and insights into host niche adaptation. In *M. humberi*, 10,633 genes were predicted by the genome annotation, of which 92.0% have putative functions assigned to them, and roughly 17% of the genome was marked as repetitive sequences. They discovered that 18.5% of the *M. humberi* genome resembles proteins linked to pathogen host interaction, while the *M. humberi* strain ESALQ1638 genome showed some distinct features that set it apart from the genomes of the other eight *Metarhizium* species. These features included a greater number of genes functionally annotated as polyketide synthases (PKSs), over-represented GO terms linked to the transport of ions, organic matter, and amino acids, a higher proportion of repetitive elements, and higher levels of RIP-induced point mutations [62]. Similarly, Binneck et al. (2019) studied whole-genome sequencing of *Metarhizium rileyi* (Hypocreales, Clavicipitaceae), strain Cep018-CH2/ARSEF 7053. Results revealed that 31,808,756 bp was the total length of the final assembly, made up of 249 scaffolds, 240 of which were larger than 1000 bp, and 1044 contigs. The longest scaffold had a length of 2,535,063 bp, while the N50 scaffold had a length of 815,204 bp, and the L50 value was 10. There was 51.30% G+C content altogether. Gene prediction and annotation showed 8945 protein codings and 102 tRNA genes. AntiSMASH analysis revealed 30 gene clusters that were involved in the biosynthesis of specialized metabolites, and functional annotation of the predicted proteins revealed key genes coding for peptidases, carbohydrate-active enzymes, secreted proteins, and transcription factors [63]. Gene annotation in the KOG, GO, KEGG, and DVDF databases yielded 7059, 9994, 3775, and 3054 genes, respectively.

*F. parceramosum* and *F. aff. Solani,* with a bootstrap support of 48, and *F. vanettenii,* with 32, indicates a close evolutionary relationship among these species. Additionally, *F. solani* KMZW-1 forms a clade with *F. falciforme* and *F. coffeatum*, with a bootstrap support of 28.2116 and 19.3965, respectively, highlighting its phylogenetic associations. The similar findings revealed the taxonomic and evolutionary position of *M. anisopliae var. anisopliae* [64]. OrthoFinder analysis revealed that *F. solani* KMZW-1 possesses 269 unique genes and shares 142 genes with *F. solani* while sharing fewer genes with other strains, indicating varying degrees of genetic divergence. All strains share a core genome of 8728 genes, representing fundamental conserved functions. These reports aligned with Guo et al. (2023), who reported the structure and specific gene categories in the *P. herquei* genome and then comprehensively compared the results with those of the two well-studied *Penicillium* species, *P. decumbens* and *P. chrysogenum*, using OrthoFinder [65]. Additionally, the collinearity analysis between the genomes of *F. solani* KMZW-1 and *F. solani-melongenae* exhibited a high degree of synteny. Most of the contigs of *F. solani* KMZW-1 correspond to the contigs of *F. solani-melongenae*. These findings provide important evidence for understanding the evolutionary relationships and ecological characteristics of *F. solani* KMZW-1.

Due to high damaging potential of *B. dorsalis*, its control often involves multiple tactics, including the use of entomopathogenic fungus. In this study, *B. dorsalis* adult females and males were treated with different conidial suspensions of the *F. solani* KMZW-1 strain at different concentrations (1 × 10^7^, 1 × 10^6^, 1 × 10^5^, 1 × 10^4^ conidia/m). After 12 days of treatment, the cumulative average mortality rates of *B. dorsalis* adults were recorded as follows: 56.67% (68.89%), 52.22% (58.89%), 44.44% (53.33%), and 34.44% (46.67%) for females (males), respectively, compared to a survival rate of 85.56% (80%) in the control group. The calculated LogLC50 for *F. solani* KMZW-1 against female and male *B. dorsalis* adults was 5.662 and 4.486, respectively. Furthermore, after treatment with high concentrations (1 × 10^11^, 1 × 10^10^, 1 × 10^9^, 1 × 10^8^ conidia/mL) of *F. solani* KMZW-1, the cumulative average mortality rates of *B. dorsalis* adults were significantly higher than the control 14.44% (20%) after 12 days, reaching 67.78% (75.56%), 67.78% (75.56%), 65.56% (77.78%), and 58.89% (76.67%), respectively.

These findings are consistent with the results of many other researchers assessing the pathogenicity of different entomopathogenic fungi against *B. dorsalis*. For example, Cherry and Moore (2006) extensively observed *M. anisopliae* infection on *B. dorsalis* and found that approximately 70% of the treated fruit fly larvae were heavily infected, with a survival rate of only 30% [66]. These results indicated that *M. anisopliae* has high pathogenicity against *B. dorsalis*, similar to the pathogenic potential observed with the *F. solani* KMZW-1 in this study. Similarly, seven different *Fusarium* species were tested against *Helicoverpa armigera* for their insecticidal potential. In field conditions, all of the collected fungi were insecticidal; however, some were severely lethal to *H. armigera* larvae. After nine days, mortality reached 91% at a concentration 1 × 10^8^ conidia/mL, while *F. solani* isolate displayed the highest toxicity against *H. armigera* larvae [67].

Jackson and Dunlap (2012) found that *Isaria fumosorosea* (Hypocreales, Cordycipita-ceae) treatment significantly reduced the survival rate of *B. dorsalis* larvae up to 40%, with an infection rate of 90% [68]. Similarly, Murtaza et al. (2022) tested the efficacy of three entomopathogenic fungi, including *M. anisopliae*, *B. bassiana,* and *V. lecanii*, against *Bactrocera zonata* (Diptera, Tephritidae) stages under different laboratory conditions. They found that *B. bassiana* and *M. anisopliae* were more pathogenic to all stages of the fruit fly as compared to *V. lecanii*. Following exposure to concentrations of 1 × 10^10^ conidia/mL of *B. bassiana*, the highest rates of mortality were observed for the third larval instar and the pupal stage, with 68.67% and 89.67%, respectively, while adults were most susceptible to all three fungus. However, at a 1 × 10^10^ conidial/mL concentration, *M. anisopliae* was more virulent against adult *B. zonata* flies as compared to *B. bassiana* and *V. lecanii* [69]. In our studies, *F. solani* KMZW-1 exhibited a concentration-dependent effect on both male and female adults of *B. dorsalis*, with a markedly higher impact on male adults. This suggests the strong potential of *F. solani* KMZW-1 for use in integrated pest management programs targeting *B. dorsalis*.

## 5. Conclusions

The fungus identified as *F. solani* KMZW-1 and its whole-genome sequencing revealed a genome size of 47,239,278 bp, comprising 27 contigs, with a GC content of 51.16% and a completeness of 97.93%. Additionally, the DFVF sequence identifier *Fusarium* 0G092560.1 and 35 gene clusters associated with secondary metabolite biosynthesis were identified. Comparative genomic analysis revealed that *F. solani* KMZW-1 possess 269 unique genes and the collinearity analysis exhibited a high degree of synteny with *F. solani-melongenae*. These findings provide crucial insights into the genetic characteristics, pathogenic mechanisms, and biocontrol potential of *F. solani* KMZW-1. Subsequently, the pathogenicity of *F. solani* KMZW-1 against *B. dorsalis* demonstrated a concentration dependent increase in mortality rates. Notably the pathogenicity of *F. solani* KMZW-1 was significantly higher in male adults compared to females with LogLC50 values of 5.662 and 4.486 for female and male *B. dorsalis* adults, respectively. These findings provide robust genomic evidence supporting the potential of *F. solani* KMZW-1 for integrated pest management strategies against *B. dorsalis*.

## Figures and Tables

**Table 1:** Genome assembly features of *Fusarium solani* KMZW-1.

Contig Length (bp)	Contig Number	Contig N50 (bp)	Contig N90 (bp)	GC Content (%)
47,239,278	27	2,751,789	1,018,923	51.16

Note: Contig length (bp) indicates the length of contig that is more than 1 kb. Contig number indicates the number of contig above 1 kb. Contig N50 (bp): length of contig N50; contig N90 (bp): length of contig N90; GC content (%): GC content; gaps number: the number of gaps.

**Table 2:** BUSCO evaluation statistics against genome of *Fusarium solani* KMZW-1.

Complete BUSCOs (C)	Complete and Single-Copy BUSCOs (S)	Complete and Duplicated BUSCOs (D)	Fragmented BUSCOs (F)	Missing BUSCOs (M)	Total Lineage BUSCOs
284 (97.93%)	281 (96.90%)	3 (1.03%)	4 (1.38%)	2 (0.69%)	290

Note: complete BUSCOs: find the complete gene number; complete and single-copy BUSCOs: the number of single-copy genes; complete and duplicated BUSCOs: number of duplicated genes; fragmented BUSCOs: predicts incomplete gene number; missing BUSCOs: no predicted number of genes; total lineage BUSCOs: the number of conserved gene sets.

**Table 3:** Gene function annotation statistics for *Fusarium solani* KMZW-1 using different databases.

Database	Number	100 ≤ Length < 300	Length ≥ 300
**GO_Annotation**	9994	2279	7603
**KEGG_Annotation**	3775	913	2788
**KOG_Annotation**	7059	1422	5575
**Pfam_Annotation**	10,510	2346	8076
**Swissprot_Annotation**	10,651	1934	8717
**TrEMBL_Annotation**	13,862	3729	9992
**nr_Annotation**	13,867	3731	9995
**All_Annotated**	13,869	3733	9995

Note: database: annotated functional database; number: the number of genes on the annotation; 100 ≤ length < 300: the number of genes whose length is 100~300 bp; length ≥ 300: the number of genes with a length greater than 300 bp.

## Data Availability

The original contributions presented in the study are included in the article/Supplementary Materials. Further inquiries can be directed to the corresponding authors.

## Supplementary Materials

The following supporting information can be downloaded at: https://www.mdpi.com/article/10.3390/cimb46100688/s1.
